# Chronic Rhinosinusitis without Nasal Polyps in Asian Patients Shows Mixed Inflammatory Patterns and Neutrophil-Related Disease Severity

**DOI:** 10.1155/2019/7138643

**Published:** 2019-01-15

**Authors:** Dae Woo Kim, Kyoung Mi Eun, Eun Youn Roh, Sue Shin, Dong-Kyu Kim

**Affiliations:** ^1^Department of Otorhinolaryngology-Head and Neck Surgery, Boramae Medical Center, Seoul National University College of Medicine, Seoul, Republic of Korea; ^2^Department of Laboratory Medicine, Boramae Medical Center, Seoul National University College of Medicine, Seoul, Republic of Korea; ^3^Department of Otorhinolaryngology-Head and Neck Surgery, Chuncheon Sacred Heart Hospital, Hallym University College of Medicine, Chuncheon, Republic of Korea; ^4^Institute of New Frontier Research, Hallym University College of Medicine, Chuncheon, Republic of Korea

## Abstract

Chronic rhinosinusitis (CRS) shows heterogeneous immunologic features. Western studies revealed that CRS without nasal polyps (CRSsNP) showed a predominantly type 1 immune response and CRS with nasal polyps (CRSwNP) was characterized by type 2 immune response; however, the detailed immunologic profile of CRSsNP in Asian patients has not been thoroughly investigated. Therefore, we investigated the inflammatory endotypes of CRSsNP in Asian patients. Patients with CRSsNP (*N* = 57), patients with CRSwNP (*N* = 13), and a control group (*N* = 10), who underwent endoscopic sinus surgery, were enrolled; uncinate process (UP) tissues were harvested from all patients. Homogenates were prepared from the UP of each group, and immunologic profiles were analyzed, including major cytokines (32 inflammatory mediators). When comparing the UPs between groups, CRSsNP patients showed higher levels of Th2 cytokines (IL-4 and IL-13), eosinophilic chemokines (CCL-11 and CCL-24), ECP, and total IgE expression than control subjects. In addition, several neutrophilic markers (IL-1*α*, IL-6, IL-8, CXCL-1, CXCL-2, and MPO), IL-17A, IL-22, and TNF-*α* were dominant in CRSsNP patients. Among these inflammatory mediators, IL-17A showed higher expression levels in CRSsNP patients than in the control group and CRSwNP patients. However, IFN-*γ* expression was not significantly elevated in CRSsNP patients. The levels of neutrophil-associated cytokines were well correlated with each other; of which, CXCL2, IL-8, and MMP-9/TIMP-1 levels were significantly correlated with disease extent (*r* = 0.338, *r* = 0.317, and *r* = 0.424, respectively). However, the levels of eosinophil-associated cytokines showed little correlation with each other and were not correlated with disease extent. Our study revealed that Asian CRSsNP patients showed a mixed (types 2 and 17) immune response, but neutrophil-related markers were dominant and associated with disease extent. Knowledge of this immunologic feature may help clinicians make better individual treatment decisions for Asian CRSsNP patients.

## 1. Introduction

Chronic rhinosinusitis (CRS) is defined as an inflammatory condition involving the paranasal sinuses and lining of the nasal passages that persists for more than 12 weeks. CRS is one of the most common diseases of the upper airway and is associated with a high risk of poor quality of life [1]. Several studies have reported its prevalence as approximately 11% in European countries, 13% in the United States, 8% in China, and 7% in South Korea [2–5]. CRS is classified into two different clinical phenotypes based on the presence or absence of nasal polyps (NPs): CRS with NPs (CRSwNP) and CRS without NPs (CRSsNP) [6]. Recently, emerging evidence has suggested that distinct immunologic mechanisms are involved in CRSsNP and CRSwNP, suggesting the presence of different immunologic endotypes [7–10]. Additionally, these distinctions appear as geographical differences in CRSwNP patients [11]. Moreover, one study revealed that second-generation Asian patients with CRSwNP living in the United States showed higher noneosinophilic characteristics; the study suggests that in these patients, noneosinophilic inflammation in CRSwNP was maintained, probably as a consequence of their genetic background [12]. Therefore, information on the inflammatory endotype is helpful in determining the clinical course of CRS patients and in decision-making for their treatment [13, 14].

Although CRSwNP patients in Western countries are characterized by Th2 inflammation and Asian patients have mainly demonstrated a Th1/Th2/Th17 mixed pattern [15–19], the immunologic profile in patients with CRSsNP is still unclear. To date, only some studies have demonstrated the immunologic characteristics of CRSsNP. In addition, the mechanism of inflammation in CRSsNP is still a topic of debate. Initially, some studies showed increased expression of interferon gamma (IFN-*γ*) and transforming growth factor- (TGF-) *β* in CRSsNP compared to CRSwNP [20, 21]. However, recent studies using the same nasal tissues have demonstrated that the IFN-*γ* level in CRSsNP patients was not significantly elevated compared with that in controls or CRSwNP patients [22–24]. Furthermore, limited studies have been published on the immunologic characteristics of CRSsNP in Asian patients [23]. One recent multicenter study described that the Th1/Th2/Th17 cytokine profiles in patients with CRSsNP showed variations among 6 regions (Adelaide, Benelux, Berlin, Beijing, Chengdu, and Tochigi) [11, 16].

Therefore, we sought to investigate the inflammatory patterns of CRSsNP in Asian patients, using the same tissues, such as the uncinate process (UP) mucosa, among the three groups (control subjects, CRSsNP patients, and CRSwNP patients).

## 2. Materials and Methods

### 2.1. Patients and Tissue Samples

Patients with CRSsNP (*N* = 57) or CRSwNP (*N* = 13) and a control group (*N* = 10), who underwent endoscopic sinus surgery, were enrolled; UP tissues were harvested from all patients. All subjects provided written informed consent for participation in the study, and the study was approved by the internal review board of the Seoul National University Hospital, Boramae Medical Center. The diagnosis of CRS was based on the 2012 European Position Paper on Rhinosinusitis and Nasal Polyps (EPOS) guidelines, which include medical history as well as physical examination, nasal endoscopy, and computed tomography findings of the sinuses [6]. Exclusion criteria were as follows: (1) age below 18 years; (2) prior treatment with antibiotics, systemic or topical corticosteroids, or other immune-modulating drugs within 4 weeks before surgery; and (3) unilateral rhinosinusitis, antrochoanal polyps, allergic fungal sinusitis, cystic fibrosis, or immotile ciliary disease. Tissues were obtained from patients without any sinonasal diseases—a control group—during other rhinologic surgeries, such as skull base, lacrimal duct, or orbital decompression surgery. Consistent with our previous work [25, 26], UP tissue was obtained from control subjects and patients with CRSsNP or CRSwNP. Each sample obtained was divided into two parts: one was fixed in 10% formaldehyde and embedded in paraffin for histological analysis and the other was immediately frozen and stored at -80°C for tissue homogenates. Paraffin-embedded nasal tissue sections (5 *μ*m) were stained with hematoxylin and eosin. Tissue eosinophilia was defined as the ratio of eosinophils to total inflammatory cells per high-power field (400x) in nasal tissue. The number of eosinophils was counted in a high-power field, in which eosinophils in the mucosa were visible as the densest cellular infiltrate beneath the epithelium. Five visual fields were examined per section to determine the mean percentage of eosinophils among inflammatory cells. Nasal samples were homogenized with a mechanical homogenizer at 1,000 rpm for 5 min on ice. After homogenization, the suspensions were centrifuged at 3,000 rpm for 10 min at 4°C and the supernatants were separated and stored at -80°C for further analysis of cytokines and other inflammatory mediators. Atopic status was evaluated by screening for serum-specific IgE antibodies to common aeroallergens using ImmunoCAP® assays (Phadia AB, Uppsala, Sweden) according to the manufacturer's recommendations. Disease extent was evaluated by using the Lund-Mackay staging system. Additional information and details of the subjects' characteristics are listed in Table 1.

### 2.2. Measurement of Inflammatory Mediators

The protein concentrations for tissue extracts were determined using the Pierce 660 nm Protein Assay Kit (Thermo Scientific Inc., NY, USA). Protein levels in tissue homogenates were normalized to the concentration of total protein (mg/mL). Samples were thawed at room temperature and vortexed to ensure well-mixed samples. In the present study, total IgE and eosinophil cationic protein (ECP) levels in the mucosal tissue were measured by ImmunoCAP®, and the sensitivities for total IgE and ECP were 2 kU/L and 2 *μ*g/L, respectively. Additionally, we used multiplex cytokine analysis kits (interleukin- (IL-) 1*α*, IL-1*β*, IL-2R*α*, IL-4, IL-5, IL-6, IL-8, IL-10, IL-13, IL-17A, IL-22, IL-23, IFN-*γ*, tumor necrosis factor- (TNF-) *α*, C-C motif chemokine- (CCL-) 11, CCL-13, CCL-24, RANTES, chemokine (C-X-C motif) ligand- (CXCL-) 1, CXCL-2, CXCL-8, myeloperoxidase (MPO), VCAM-1, ICAM-1, MMP-1 (matrix metalloproteases-1), MMP-2, MMP-3, MMP-7, MMP-9, TIMP-1 (tissue inhibitor of matrix metalloproteases-1), and TGF-*β*1), which were obtained from R&D Systems (cat. no. LMSAHM; R&D Systems Inc., Minneapolis, MN, USA) in the present study, and data were collected using Luminex 100 (Luminex, Austin, TX, USA). Sensitivities of each cytokine are as follows: IL-1*α* (0.9 pg/mL), IL-1*β* (0.8 pg/mL), IL-2R*α* (1.3 pg/mL), IL-4 (9.3 pg/mL), IL-5 (0.5 pg/mL), IL-6 (1.7 pg/mL), IL-8 (1.82 pg/mL), IL-10 (1.6 pg/mL), IL-13 (32.4 pg/mL), IL-17A (1.8 pg/mL), IL-22 (11.7 pg/mL), IL-23 (11.4 pg/mL), IL-33 (1.8 pg/mL), IFN-*γ* (0.4 pg/mL), TNF-*α* (1.2 pg/mL), CCL-11 (14.6 pg/mL), CCL-13 (0.42 pg/mL), CCL-24 (1.34 pg/mL), RANTES (1.8 pg/mL), CXCL-1 (5.3 pg/mL), CXCL-2 (7.86 pg/mL), MPO (20.4 pg/mL), VCAM-1 (238 pg/mL), ICAM-1 (87.9 pg/mL), MMP-1 (2.7 pg/mL), MMP-2 (108 pg/mL), MMP-3 (5.3 pg/mL), MMP-7 (23.2 pg/mL), MMP-9 (13.6 pg/mL), TIMP-1 (3.42 pg/mL), and TGF-*β*1 (2.1-24.6 pg/mL). Data analysis was performed using the MasterPlex QT version 2.0 (MiraiBio, Alameda, CA, USA). All assays were run in duplicate according to the manufacturer's protocol. In addition, we used the ratio of ECP/MPO based on protein concentrations for tissue extracts.

### 2.3. Statistical Analysis

Statistical analyses were performed using the IBM SPSS 21 (IBM Inc., Armonk, NY, USA) and GraphPad Prism software 6.0 (GraphPad Software Inc., La Jolla, CA, USA). For comparisons among multiple groups, the Kruskal-Wallis test was first used to establish a significant difference and then if significance was detected, the Mann-Whitney *U* test was performed between the two groups and Bonferroni correction was used to adjust the significance level for each comparison. Correlations were assessed by Spearman's rank correlation. The significance level was set at an *α* value of 0.05.

## 3. Results

**Figure 1:**
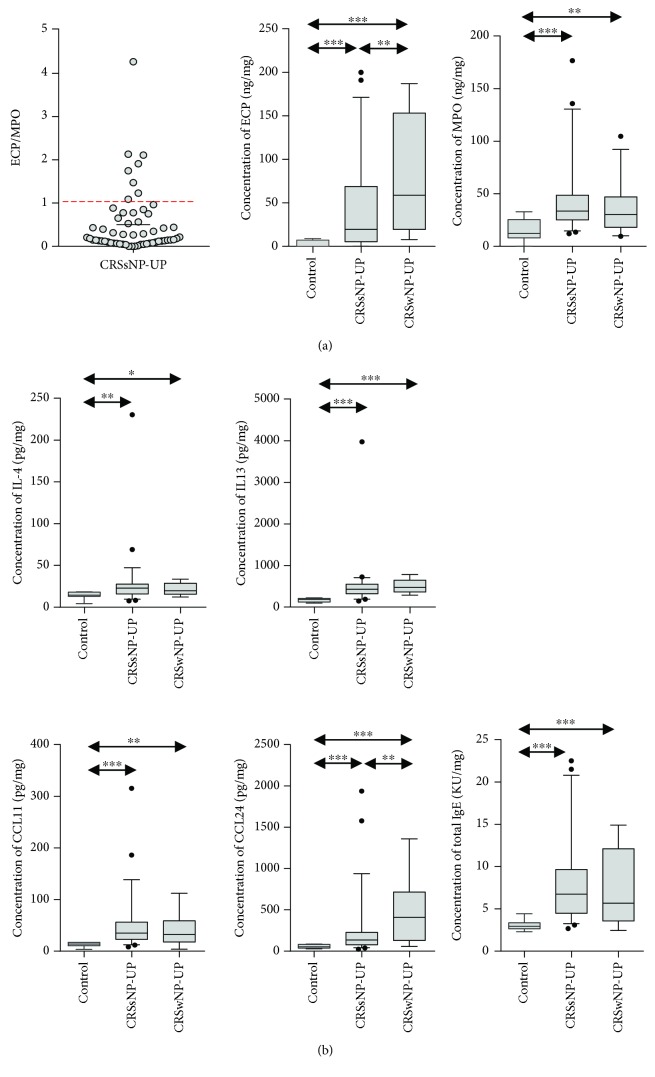
Ratio of ECP to MPO level and expression of Th2-related mediators. (a) Concentration of ECP and MPO (ECP/MPO) and (b) concentration of IL-4, IL-13, CCL-11, CCL-24, and total IgE. ECP: eosinophil cationic protein; MPO: myeloperoxidase.

Among 32 inflammatory mediators, only those that showed statistically significant results are presented in the figures. First, to classify the populations according to the histological subtype in the CRSsNP patients, we divided these patients according to tissue eosinophilia: none, ≤5%, >5 to ≤10%, and >10%. We found that the most common histologic type of CRSsNP was ≤5% tissue eosinophilia (38%), followed by none (34%), >10% tissue eosinophilia (15%), and >5 to ≤10% tissue eosinophilia (13%). The results regarding the ratio of ECP to MPO were as follows: 30% of all CRSsNP patients showed ratios ≤0.1, 42% showed ratios >0.1 and ≤0.5, 14% showed ratios >0.5 and ≤1, and 14% showed ratios >1 (Figure 1(a)). When comparing UPs among groups, CRSsNP and CRSwNP patients showed higher expression levels of ECP and MPO than did the control subjects (Figure 1(a)). In addition, ECP expression was significantly higher in patients with CRSwNP than in those with CRSsNP, whereas there was no significant difference in MPO expression between CRSsNP and CRSwNP patients. The patterns of Th2 immune response in CRSsNP patients, compared to those of the control group and CRSwNP patients, are presented in Figure 1(b). The expression of Th2 cytokines, such as IL-4 and IL-13, was higher in CRSsNP and CRSwNP patients than in control subjects. Additionally, some eosinophilic recruiting markers (CCL-11 and CCL-24) and total IgE showed higher levels in both CRSsNP and CRSwNP patients than in control subjects. However, there was no significant difference in these cytokines and chemokines between CRSsNP and CRSwNP patients, except for CCL-24 (Figure 1(b)). The CCL24 expression was higher in CRSwNP patients than in CRSsNP patients.

**Figure 2:**
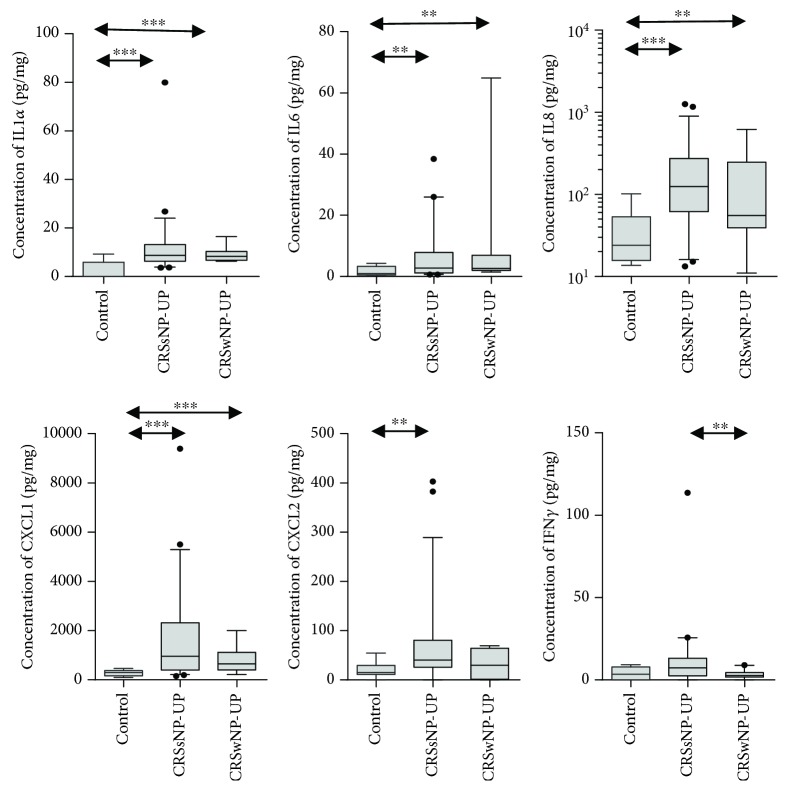
Expression of neutrophilic and Th1-related mediators; concentration of IL-1*α*, IL-6, IL-8, CXCL-1, CXCL-2, and IFN-*γ*.

**Figure 3:**
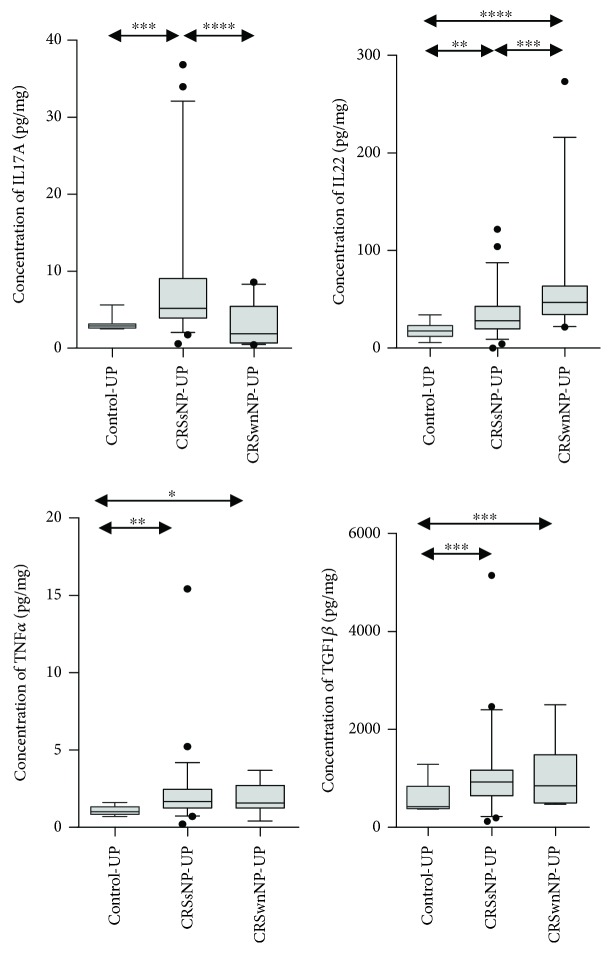
Expression of Th17- and Treg-related mediators; concentration of IL-17A, IL22, TNF-*α*, and TGF-*β*1.

**Figure 4:**
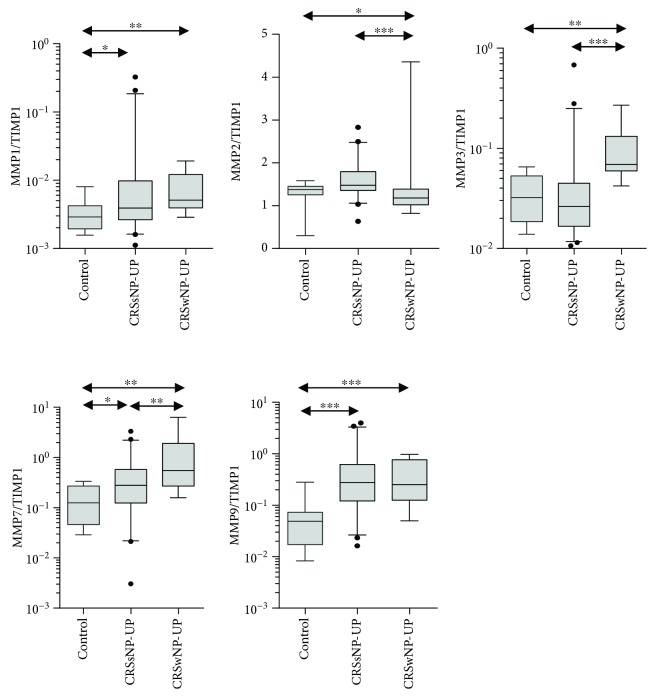
Expression of various remodeling markers; ratio of MMP-1/TIMP-1, MMP-2/TIMP-1, MMP-3/TIMP-1, MMP-7/TIMP-1, and MMP-9/TIMP-1.

Next, we investigated several inflammatory markers, which were related to neutrophilic inflammation (Figure 2). Among them, IL-1*α*, IL-6, IL-8, and CXCL-1 showed significantly higher levels in CRSsNP and CRSwNP patients than in control subjects. Moreover, CXCL-2 level was significantly increased in only CRSsNP patients. Interestingly, we observed that there was no difference in IFN-*γ* protein level between CRSsNP patients and control subjects; however, IFN-*γ* expression was significantly elevated in patients with CRSsNP compared to those with CRSwNP. Furthermore, increased levels of IL-17A, IL-22, and TNF-*α* were observed in CRSsNP patients compared to control subjects (Figure 3). Among those, IL-17A showed a significantly higher expression in CRSsNP than in CRSwNP patients. Additionally, CRSsNP patients showed higher TGF-*β*1 protein concentration than control subjects, but there was no significant difference in this parameter between CRSsNP and CRSwNP patients. We also investigated the pattern of remodeling markers in CRS (Figure 4). CRSsNP and CRSwNP patients showed increased expression of MMP-1/TIMP-1, MMP-7/TIMP-1, and MMP-9/TIMP-1, compared with that in control subjects. Specifically, MM-P1/TIMP-1 and MMP-9/TIMP-1 showed no significant differences between CRSsNP and CRSwNP patients. In the analysis of adhesion molecules, there was no significant difference between the three groups.

**Figure 5:**
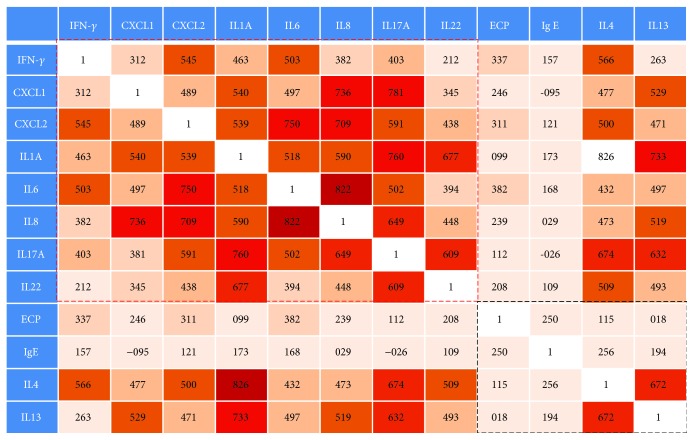
Analysis of correlation between several representative Th1/Th2/Th17 cytokine expressions. The numbers in the boxes refer to *R* values.

To confirm the dominant immune response of CRSsNP, we performed a correlation analysis for each inflammatory mediator. In these analyses, we observed an overall higher correlation between the neutrophil-associated cytokines and chemokines (red box in Figure 5), compared with eosinophil-associated cytokines and chemokines (black box in Figure 5). Furthermore, only CXCL-2, CXCL-8, and MMP-9/TIMP-1 were significantly correlated with disease extent in CRSsNP patients (*r* = 0.338, *r* = 0.317, *r* = 0.424, respectively).

## 4. Discussion

The immunologic characteristics of CRSsNP in Asian patients have been rarely investigated. Therefore, this study investigated the inflammatory patterns among control subjects, CRSsNP patients, and CRSwNP patients in a Korean population. Recently, one study revealed that CRSsNP is a heterogeneous disease and the overall frequency of type 2 inflammation is higher than that of type 1 inflammation in the US-based population [23, 24]. In contrast with previous studies [20, 21], this study performed an analysis of the immune profile, using the same type of nasal tissues from control subjects, patients with CRSsNP, and patients with CRSwNP. The category of nasal tissue samples from each CRS population may strongly affect the result because CRS is a remarkably heterogeneous disease. Thus, using the same nasal tissues is important; if we use different nasal tissues, it would be unclear whether the difference in the immune profiles was due to differences in the anatomy of the sampled tissue or phenotype of CRS. Therefore, in the present study, we evaluated and compared the inflammatory profile between each group, using the same nasal tissues, that is, the UP tissues.

In the present study, we found relatively less tissue eosinophilia and a lower ratio of ECP/MPO in CRSsNP patients; however, Th2 cytokines (IL-4 and IL-13), eosinophilic chemokines (CCL-11 and CCL-24), ECP, and total IgE were significantly increased in CRSsNP patients, compared with those in control subjects. Further, in our study, we observed that the IFN-*γ* protein level was not significantly elevated in CRSsNP patients, but the TGF-*β*1 protein concentration was increased, compared with that in control subjects. Consistent with our findings, prior recent studies on Western patients demonstrated that IFN-*γ* was not elevated in CRSsNP compared with control subjects [23, 24]. Moreover, we found that patients with CRSsNP showed prominent neutrophilic inflammation with upregulation of proinflammatory cytokines, including IL-1*α*, IL-6, IL-8, CXCL-1, CXCL-2, and MPO. Furthermore, IL-17A, IL-22, and TNF-*α* expression levels were higher in CRSsNP patients than in control subjects. Among those, only IL-17A is the most prominent in CRSsNP, compared to that in control and CRSwNP patients. Collectively, these data indicate that patients with CRSsNP show an increased mixed Th cell (Th2/Th17) immune response with increased neutrophilic inflammation.

A previous study reported that fibrocytes stimulated with IL-17A in asthma patients released proinflammatory factors that may promote neutrophil recruitment [27]. Thus, we initially hypothesized that, although CRSsNP shows mixed inflammation, the neutrophil-related inflammation may be the major pathophysiology rather than eosinophil-related inflammation in patients with CRSsNP. For these reasons, to investigate the dominant immune response of CRSsNP, we performed correlation analyses for various inflammatory mediators. In these analyses, we observed an overall higher correlation between the neutrophil-associated mediators and chemokines (red box in Figure 5), compared with that of eosinophil-associated mediators and chemokines (black box in Figure 5). Moreover, we observed a correlation between disease severity and some neutrophil-associated mediators (CXCL-2 and IL-8). However, all eosinophil-associated mediators do not display a correlation with disease extent. In contrast with our findings, Western studies suggest that Th2 immune response was a major inflammatory pattern in CRSsNP [22–24]. We thought that this discrepancy might be due to genetic factors.

On the analysis of the remodeling markers, MMP-1/TIMP-1, MMP-7/TIMP-1, and MMP-9/TIMP-1 showed higher expression in CRSsNP patients than in control subjects. We used the ratio of MMP/TIMP, because the activity of MMP-9 is regulated by TIMP-1. MMPs have proteolytic activity and a strong capability to degrade extracellular matrix components and are widely accepted to play a role in breakdown of collagen. Thus, in this study, overall expression of MMPs was higher in CRSwNP than in CRSsNP patients. However, there was no difference in MMP-1/TIMP-1 and MMP-9/TIMP-1 expression between CRSsNP and CRSwNP. Among these, MMP-9 especially has been reported to be associated with neutrophilic inflammation [28–30]. In addition, macrophages form the main source of MMP-9 in normal lungs, but neutrophils secrete MMP-9 in chronic obstructive lung disease [31]. Moreover, the concentration and activity of MMP-9 correlates with the disease severity of chronic obstructive lung disease [32]. In this study, we also found that the ratio MMP-9/TIMP-1 correlated positively with CRSsNP severity.

Interestingly, our recent study proposed a two-track treatment strategy according to the clinical scoring system for CRS [13], and that study recommended that non-Th2 CRS patients (less eosinophilic) be managed with sufficient antibiotic therapies, including a long-term macrolide followed by surgery or by applying newly emerging anti-type 17 biologic agents [13]. Meanwhile, in the present study, we found that neutrophilic inflammation played an important role in Asian CRSsNP patients. Therefore, we thought that the therapeutic strategy for Asian CRSsNP patients, who are characterized by a neutrophil-dominant immune response, may be similar to that for non-Th2 CRS patients. However, this study has some limitations. The most common sites of origin for nasal polyposis are UP tissues, but sometimes they are destroyed by nasal polyposis. Thus, the number of UP tissues from CRSwNP patients was smaller than that from CRSsNP patients in this study. For this reason, we could not perform a comparison of immunologic features according to the endotypes of CRSwNP, such as the eosinophilic and noneosinophilic types.

## 5. Conclusions

In the present study, Asian patients with CRSsNP showed mixed inflammatory profiles, but the neutrophil-related markers were dominant and associated with disease extent. Our study findings may be helpful in providing novel information on inflammatory endotypes of CRSsNP in Asian populations, which could aid the development of personalized therapeutic strategies.

## Figures and Tables

**Table 1:** Characteristics of the study population.

	Control (*N* = 10)	CRSsNP (*N* = 57)	CRSwNP (*N* = 13)
Tissue used	UP	UP	UP
Male/female	3/7	40/17	6/7
Age (year) (SD)	50 (12)	48 (17)	50 (13)
Atopy (%)	1 (10%)	16 (28%)	5 (38%)
Asthma (%)	0 (0%)	2 (4%)	4 (31%)
CT scores	NA	9.5 (4.4)	12.5 (4.4)

## Data Availability

The datasets generated and/or analyzed during the current study are not publicly available due to the policy of Chuncheon Sacred Heart Hospital, but are available from the corresponding author on reasonable request.
